# Memantine ameliorates motor impairments and pathologies in a mouse model of neuromyelitis optica spectrum disorders

**DOI:** 10.1186/s12974-020-01913-2

**Published:** 2020-08-11

**Authors:** Leung-Wah Yick, Chi-Ho Tang, Oscar Ka-Fai Ma, Jason Shing-Cheong Kwan, Koon-Ho Chan

**Affiliations:** 1 Department of Medicine, Li Ka Shing Faculty of Medicine, The University of Hong Kong, Hong Kong, Hong Kong; 2 Neuroimmunology and Neuroinflammation Research Laboratory, Li Ka Shing Faculty of Medicine, The University of Hong Kong, Hong Kong, Hong Kong; 3 Department of Medicine, The University of Hong Kong, 4/F, Professorial Block, Queen Mary Hospital, 102 Pokfulam Road, Hong Kong, Hong Kong

**Keywords:** Aquaporin-4, Autoimmunity, Memantine, Neuroinflammation, Neuromyelitis optica

## Abstract

**Background:**

Neuromyelitis optica spectrum disorders (NMOSD) are central nervous system (CNS) autoimmune inflammatory demyelinating diseases characterized by recurrent episodes of acute optic neuritis and transverse myelitis. Aquaporin-4 immunoglobulin G (AQP4-IgG) autoantibodies, which target the water channel aquaporin-4 (AQP4) on astrocytic membrane, are pathogenic in NMOSD. Glutamate excitotoxicity, which is triggered by internalization of AQP4-glutamate transporter complex after AQP4-IgG binding to astrocytes, is involved in early NMOSD pathophysiologies. We studied the effects of memantine, a N-methyl-D-aspartate (NMDA) receptor antagonist, on motor impairments and spinal cord pathologies in mice which received human AQP4-IgG.

**Methods:**

Purified IgG from AQP4-IgG-seropositive NMOSD patients were passively transferred to adult C57BL/6 mice with disrupted blood-brain barrier. Memantine was administered by oral gavage. Motor impairments of the mice were assessed by beam walking test. Spinal cords of the mice were assessed by immunofluorescence and ELISA.

**Results:**

Oral administration of memantine ameliorated the motor impairments induced by AQP4-IgG, no matter the treatment was initiated before (preventive) or after (therapeutic) disease flare. Memantine profoundly reduced AQP4 and astrocyte loss, and attenuated demyelination and axonal loss in the spinal cord of mice which had received AQP4-IgG. The protective effects of memantine were associated with inhibition of apoptosis and suppression of neuroinflammation, with decrease in microglia activation and neutrophil infiltration and reduction of increase in levels of proinflammatory cytokines including interleukin-1β (IL-1β), interleukin-6 (IL-6), and tumor necrosis factor-α (TNF-α). In addition, memantine elevated growth factors including brain-derived neurotrophic factor (BDNF), glial cell line-derived neurotrophic factor (GDNF), and vascular endothelial growth factor (VEGF) in the spinal cord.

**Conclusions:**

Our findings support that glutamate excitotoxicity and neuroinflammation play important roles in complement-independent pathophysiology during early development of NMOSD lesions, and highlight the potential of oral memantine as a therapeutic agent in NMOSD acute attacks.

## Introduction

Neuromyelitis optica spectrum disorders (NMOSD) are inflammatory demyelinating disorders of the central nervous system (CNS) clinically characterized by recurrent attacks of acute optic neuritis, transverse myelitis, and less frequently encephalitis. Patients with severe NMOSD can develop blindness, paraplegia, and even mortality [1]. The majority of NMOSD patients are seropositive for aquaporin-4 immunoglobulin G autoantibodies (AQP4-IgG), which target the water channel aquaporin-4 (AQP4) [2, 3] that is highly expressed on the membrane of astrocytic foot processes [4]. NMOSD seropositive for AQP4-IgG are considered as an autoimmune astrocytopathy [5, 6].

Diverse lesion pathologies have been observed in the CNS of NMOSD patients, suggesting that multiple pathophysiologies are involved in acute attacks of NMOSD including both complement-dependent and -independent mechanisms [7]. Notably, the initiator of complement cascade, C1q, is absent in the quiescent CNS. Complement-independent events likely contribute to the early development of NMOSD lesions. These events include AQP4 loss from internalization and degradation of the antigen-autoantibody complex [8], glutamate excitotoxicity from reduction of the glutamate transporter excitatory amino acid transporter 2 (EAAT2) [9, 10], neuroinflammation due to release of proinflammatory cytokines and chemokines from astrocytes [11], and antibody-dependent cell-mediated cytotoxicity (ADCC) which involves microglia/macrophages and granulocytes [12].

Our recent study showed that mice which received IgG from AQP4-IgG-seropositive NMOSD patients developed complement-independent spinal cord pathologies, including AQP4 and glial fibrillary acidic protein (GFAP) loss, EAAT2 decrease, microglia activation, neutrophil infiltration, demyelination, and axonal loss, which were associated with motor impairments [13]. These findings support that AQP4-IgG binding to astrocytes triggers glutamate excitotoxicity secondary to EAAT2 decrease, neuroinflammation, and astrocyte cytotoxicity through ADCC by activated microglia and infiltrated granulocytes. Targeting glutamate excitotoxicty caused by AQP4-IgG binding to astrocytes may be a potential therapeutic intervention in acute attacks of NMOSD.

Memantine (1-amino-3,5-dimethyladamantane hydrochloride) is a non-competitive N-methyl-D-aspartate (NMDA) receptor antagonist. It is an approved drug for the treatment of dementia in Alzheimer’s disease and Parkinson’s disease [14, 15]. Memantine has been shown to be neuroprotective in different animal models of CNS damages. In an experimental autoimmune encephalomyelitis model with optic neuritis, memantine reduced optic nerve demyelination and protected axons and retinal ganglion cells [16]. In experimental glaucomatous neurodegeneration, memantine prevented astrocytic dysfunction of the optic nerves by enhancing mitochondrial fission, increasing mitochondrial volume density and length, and reducing auto-phagosome formation [17]. Following subarachnoid hemorrhage, memantine suppressed apoptotic cascade via reducing neuronal nitric oxide synthase expression, peroxynitrite formation, and subsequent oxidative stress [18]. In this study, we investigated if memantine exerts neuroprotective effects in mice which received passive transfer of IgG from AQP4-IgG-seropositive NMOSD patients.

## Materials and methods

### Patient samples and IgG purification

Sera/plasma were obtained from 13 NMOSD patients who were AQP4-IgG-seropositive as detected by cell-based indirect immunofluorescence assay [19] and 3 healthy subjects. IgG from sera or plasma was isolated using HiTrap Protein G Sepharose columns (GE Healthcare Bio-sciences, USA). Samples were further purified with Slide-A-Lyzer Dialysis Cassettes (Thermo Scientific, USA) and concentrated with Amicon Ultra-15 centrifugal filters (Merck Millipore, Germany). Protein concentration was measured by Bradford assay (Bio-Rad, USA). Pooled IgG isolated from AQP4-IgG-seropositive NMOSD patients was termed IgG(AQP4+). Pooled IgG isolated from healthy subjects was termed IgG(con).

### Mice

Female wild-type C57BL/6 mice of age 6–8 weeks were used. Mice were housed in the animal facilities at the Laboratory Animal Unit of The University of Hong Kong. They were maintained in groups of five per cage under a 12 h dark/light cycle and provided with free access of water and chow.

### Disruption of BBB and passive transfer of IgG from NMOSD patients

**Fig. 1:**
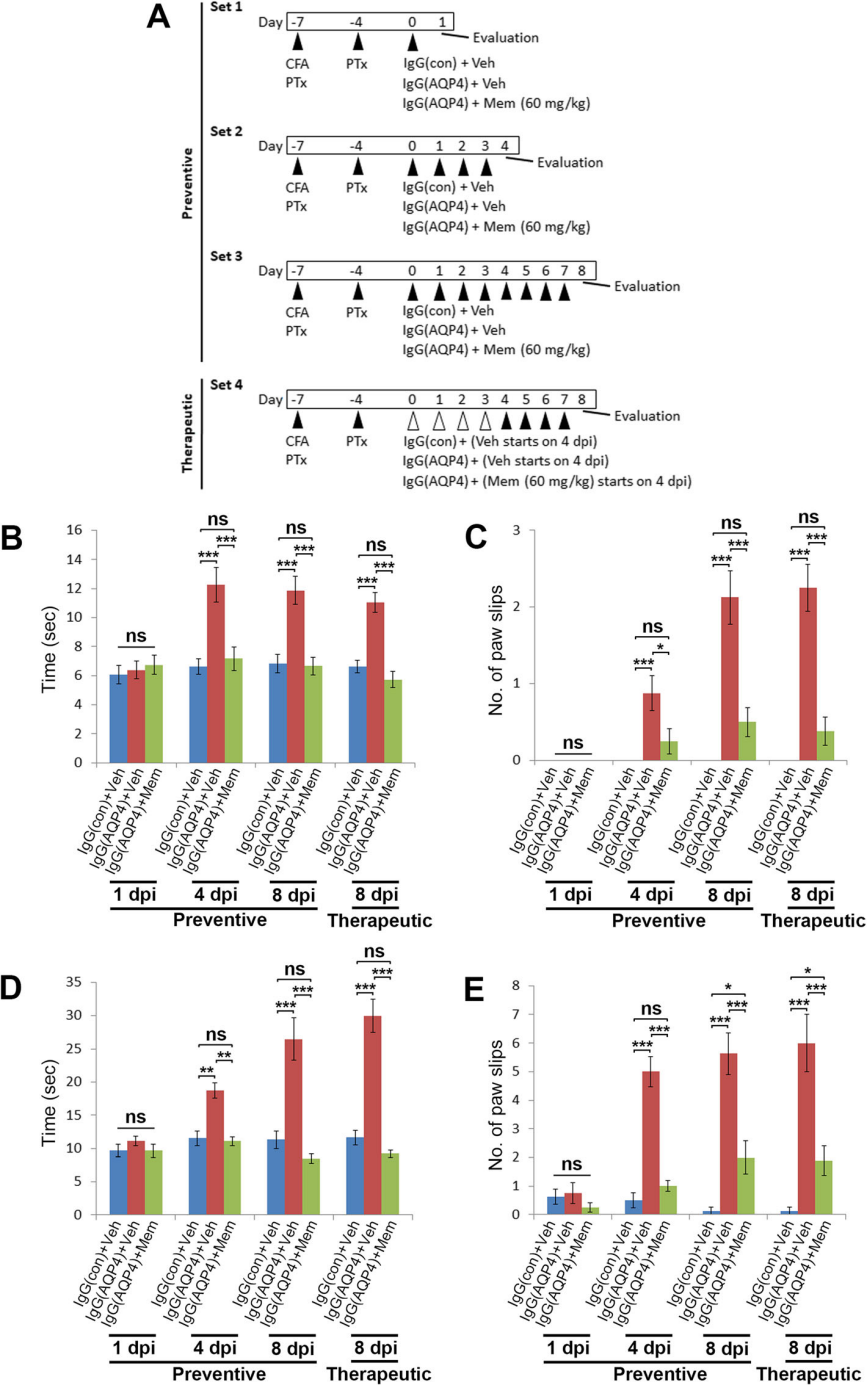
Oral administration of memantine ameliorates human AQP4-IgG-induced motor impairments in mice. **a** Animal groups and experimental procedures. **b**, **c** Time required (**b**) and number of paw slips (**c**) during walking across a 1.2 × 80 cm (width × length) beam in beam walking test on IgG(AQP4+) mice treated with preventive memantine or vehicle at 1, 4, and 8 days post-injection (dpi) or with therapeutic memantine or vehicle at 8 dpi. IgG(con) mice treated with vehicle were used as a sham control. **d**, **e** Time required (**d**) and number of paw slips (**e**) during walking across a 0.6 × 80 cm (width × length) beam in beam walking test on mice in different groups. *n* = 8 per group. Data are mean ± SEM. One-way ANOVA with Tukey-Kramer post hoc test. ns, not significant; *p < 0.05; **p < 0.01; ***p < 0.001

Animal procedures are summarized in Fig. 1a. To disrupt the BBB, mice were anaesthetized with intraperitoneal (i.p.) injection of ketamine (100 mg/kg) and xylazine (10 mg/kg). They received subcutaneous injections of complete Freund’s adjuvant (CFA, BD Biosciences, USA) containing heat-killed H37Ra *Mycobacterium tuberculosis* (Difco, USA) at 4 sites (50 μg in 50 μl CFA each site) on the hind flank on 7 days before IgG transfer. In addition, mice received i.p. injections of pertussis toxin (PTx, 200 ng in 0.2 ml PBS, List Biological Laboratories, USA) on 7 and 3 days prior to IgG transfer.

### Drug treatments

Memantine (Sigma-Aldrich, USA) was prepared by adding the compound to corn oil (Sigma-Aldrich, USA). The mixture was sonicated at room temperature for 20 min. It was freshly made immediately before each administration. Following BBB disruption, mice were randomly assigned to four sets of experiments. For preventive treatment, IgG(AQP4+) mice in set 1 received i.p. injection of 4 mg purified IgG from AQP4-IgG-seropositive NMOSD patients and oral gavage of vehicle or 60 mg/kg body weight memantine on the day of IgG passive transfer (day 0). Animals were sacrificed at 1 day post-injection (dpi). In set 2, beginning from day 0, IgG(AQP4+) mice received daily i.p. injection of purified IgG from AQP4-IgG-seropositive NMOSD patients and oral gavage of vehicle or memantine till 3 dpi. Animals were sacrificed at 4 dpi. In set 3, beginning from day 0, IgG(AQP4+) mice received daily i.p. injection of purified IgG from AQP4-IgG-seropositive NMOSD patients and oral gavage of vehicle or memantine till 7 dpi. Animals were sacrificed at 8 dpi. For therapeutic treatment in set 4, IgG(AQP4+) mice received daily i.p. injection of purified IgG from AQP4-IgG-seropositive NMOSD patients beginning from day 0 till 7 dpi. Oral gavage of vehicle or memantine was begun on the 4 dpi and continued once daily immediately after IgG injection till 7 dpi. Animals were sacrificed at 8 dpi. IgG(con) mice which received vehicle were used as sham controls in all sets of experiments. The dose of memantine was adopted from a previous study [16].

### Detection of motor impairments

Motor impairments were detected using beam walking test, in which examines the animal’s ability to keep upright and walk across an elevated narrow beam to a platform [20]. The apparatus consisted of 80-cm-long beams with width of 1.2 or 0.6 cm, resting 50 cm above a table top on two stands. Prior to CFA and PTx treatment, mice were trained for 2 days, with 3 trials a day, with walking across each of the 1.2- and 0.6-cm-wide beams. One day after completion of IgG transfer and drug treatment, the time for the mice to cross each beam and the number of paw slips during the process were recorded. A slip is defined as one or both feet coming off the top of the beam [20]. The investigators were blinded to the experimental groups during beam walking test.

### Tissue preparation and immunofluorescence

After beam walking test, mice were sacrificed by pentobarbital overdose and received intracardiac perfusion with ice-cold PBS and paraformaldehyde. Cervical spinal cords were harvested, and sectioned at 10-μm thickness with a cryostat (Thermo Fisher Scientific, USA). Immunofluorescence was performed with standard procedure. The following primary antibodies were used: (1) rabbit anti-human IgG (1:600, Dako, USA), (2) rabbit anti-AQP4 (1:200, Sigma-Aldrich, USA), (3) mouse anti-glial fibrillary acidic protein (GFAP, 1:200, Santa Cruz Biotechnology, USA), (4) goat anti-myelin basic protein (MBP, 1:200, Dako, USA), (5) rabbit anti-neurofilament heavy polypeptide (NF-H, 1:400, Sigma-Aldrich, USA), (6) rabbit anti-NR2B (1:200, Abcam, UK), (7) rabbit anti-ionized calcium-binding adapter molecule 1 (Iba-1, 1:200, Wako, Japan), (8) rat anti-lymphocyte antigen 6 complex locus G6D (Ly6G, 1:400, Abcam, UK), (9) rat anti-CD4 (1:100, Santa Cruz Biotechnology, USA), and (10) rat anti-CD8 (1:100, Santa Cruz Biotechnology, USA). Sections were then incubated with the appropriate Alexa-Fluor-conjugated secondary antibodies (Thermo Fisher Scientific, USA) at room temperature for 1 h. They were counterstained with DAPI and mounted with anti-fade reagent (Thermo Fisher Scientific, USA).

### Image processing and quantification

Measurement of immunofluorescent intensity was performed on eight rostral-to-caudal alternate cross sections of the ventrolateral white matter of the cervical spinal cord. All signals were captured with the same microscope (Nikon Eclipse Ni, Japan) and digitized with SPOT software 5.0 (Diagnostic Instruments, USA) in identical settings. Signal intensity was quantified using ImageJ software (Wayne Rasband, NIH, USA).

### Histochemistry

Luxol fast blue (LFB) staining was performed according to the manufacturer’s instructions (Abcam, UK). Briefly, spinal cord sections were rehydrated and incubated in 0.1% Luxol fast blue solution at 40 ^o^C overnight. Excess stain was rinsed with 95% ethanol. Slides were washed in distilled water, immersed in 0.05% lithium carbonate solution for 30s, and then dehydrated in serial ethanols and mounted in Permount mounting medium (Sigma-Aldrich, USA). In addition, selected sections were stained with hematoxylin and eosin (H&E) using standard procedure.

### Detection of apoptotic cells

Terminal deoxynucleotidyl transferase dUTP nick-end labeling (TUNEL) staining was performed using the In-situ Cell Death Detection Kit (Roche, Germany) according to the manufacturer’s instructions. Sections were mounted by counterstained with DAPI and mounted with anti-fade reagent (Thermo Fisher Scientific, USA). To quantify apoptotic cells, cells positive for TUNEL with condensed nuclei were counted on eight rostral-to-caudal alternate cross sections of the ventrolateral white matter of the cervical spinal cord.

### ELISA for cytokines and growth factors

Following transcardiac perfusion with ice-cold PBS, spinal cords of mice were harvested and homogenized using a lysis buffer containing protease inhibitor cocktail and phosphatase inhibitor. Levels of proinflammatory cytokines including interleukin-1β (IL-1β; RayBiotech, USA), interleukin-6 (IL-6; RayBiotech, USA) and tumor necrosis factor-α (TNF-α; RayBiotech, USA), and growth factors including brain-derived neurotrophic factor (BDNF; MyBioSource, USA), glial cell line-derived neurotrophic factor (GDNF; MyBioSource, USA) and vascular endothelial growth factor (VEGF; MyBioSource, USA) in the homogenates were determined using mouse ELISA kits according to the manufacturer’s instructions.

### Statistical analysis

Differences between groups were compared by one-way analysis of variance (ANOVA) followed by Tukey-Kramer post hoc test. Data are shown as mean ± SEM. Levels of significance are indicated with *p < 0.05, **p < 0.01, and ***p < 0.001. Calculation was performed using IBM SPSS Statistics Version 24 software.

## Results

### Memantine improved AQP4-IgG-induced motor impairments

Passive transfer of human AQP4-IgG to mice with disrupted BBB causes motor impairments [13]. To assess whether memantine improved motor performance in this animal model, beam walking test was performed in IgG(AQP4+) mice which had received either preventive or therapeutic treatment of memantine. We found that 60 mg/kg/day of memantine is effective in improving clinical severity, and therefore this dose was used in subsequent experiments (Additional file 1 a–d). H&E staining revealed no infiltration of inflammatory cells in hind limb muscles of mice in different groups; hence, no AQP4-IgG-induced myositis in hind limbs of mice contributing to motor impairments (Additional file 1e).

On a 1.2 × 80 cm (width × length) beam, no significant differences on motor performance were observed between sham, vehicle-treated, and memantine-treated mice at 1 dpi. Onset of motor impairments was found at 4 dpi, when vehicle-treated IgG(AQP4+) mice required significantly longer time to cross the beam and displayed significantly more paw slips during walking on the beam than sham mice. Preventive treatment with memantine at 60 mg/kg/day, when it was begun on the same day of IgG(AQP4+) passive transfer, completely prevented the development of motor impairments in terms of time to cross the beam and number of paw slips during walking on the beam. Therapeutic treatment with memantine at 60 mg/kg/day, begun at 4 days after the first IgG(AQP4+) passive transfer, also prevented the development of motor impairments. The efficacy of therapeutic treatment was comparable to that of preventive treatment (Fig. 1b, c, Additional files 7, 8, 9, 10). Similar findings were observed in walking test using a narrower 0.6 × 80 cm (width × length) beam that allowed detection of more subtle motor impairments. Onset of motor impairments was observed at 4 dpi. Both preventive and therapeutic treatment with memantine significantly reduced the time to cross the beam and the number of paw slips during walking on the beam (Fig. 1d, e, Additional files 11, 12, 13, 14). These findings indicated that memantine markedly ameliorated motor impairments triggered by human AQP4-IgG in mice.


**Additional files 7.** Representative video clip showing beam walking test of mouse received IgG(con) and vehicle, IgG(AQP4+) and vehicle, IgG(AQP4+) and preventive memantine, and IgG(AQP4+) and therapeutic memantine on the 1.2 x 80 cm beam


**Additional file 8.** Representative video clip showing beam walking test of mouse received IgG(con) and vehicle, IgG(AQP4+) and vehicle, IgG(AQP4+) and preventive memantine, and IgG(AQP4+) and therapeutic memantine on the 1.2 x 80 cm beam


**Additional file 9.** Representative video clip showing beam walking test of mouse received IgG(con) and vehicle, IgG(AQP4+) and vehicle, IgG(AQP4+) and preventive memantine, and IgG(AQP4+) and therapeutic memantine on the 1.2 x 80 cm beam


**Additional file 10.** Representative video clip showing beam walking test of mouse received IgG(con) and vehicle, IgG(AQP4+) and vehicle, IgG(AQP4+) and preventive memantine, and IgG(AQP4+) and therapeutic memantine on the 1.2 x 80 cm beam


**Additional file 11.** Representative video clip showing beam walking test of mouse received IgG(con) and vehicle, IgG(AQP4+) and vehicle, IgG(AQP4+) and preventive memantine, and IgG(AQP4+) and therapeutic memantine on the 0.6 x 80 cm beam


**Additional file 12.** Representative video clip showing beam walking test of mouse received IgG(con) and vehicle, IgG(AQP4+) and vehicle, IgG(AQP4+) and preventive memantine, and IgG(AQP4+) and therapeutic memantine on the 0.6 x 80 cm beam


**Additional file 13.** Representative video clip showing beam walking test of mouse received IgG(con) and vehicle, IgG(AQP4+) and vehicle, IgG(AQP4+) and preventive memantine, and IgG(AQP4+) and therapeutic memantine on the 0.6 x 80 cm beam


**Additional file 14.** Representative video clip showing beam walking test of mouse received IgG(con) and vehicle, IgG(AQP4+) and vehicle, IgG(AQP4+) and preventive memantine, and IgG(AQP4+) and therapeutic memantine on the 0.6 x 80 cm beam.

### Memantine decreased AQP4-IgG-induced AQP4 and GFAP loss

**Fig. 2:**
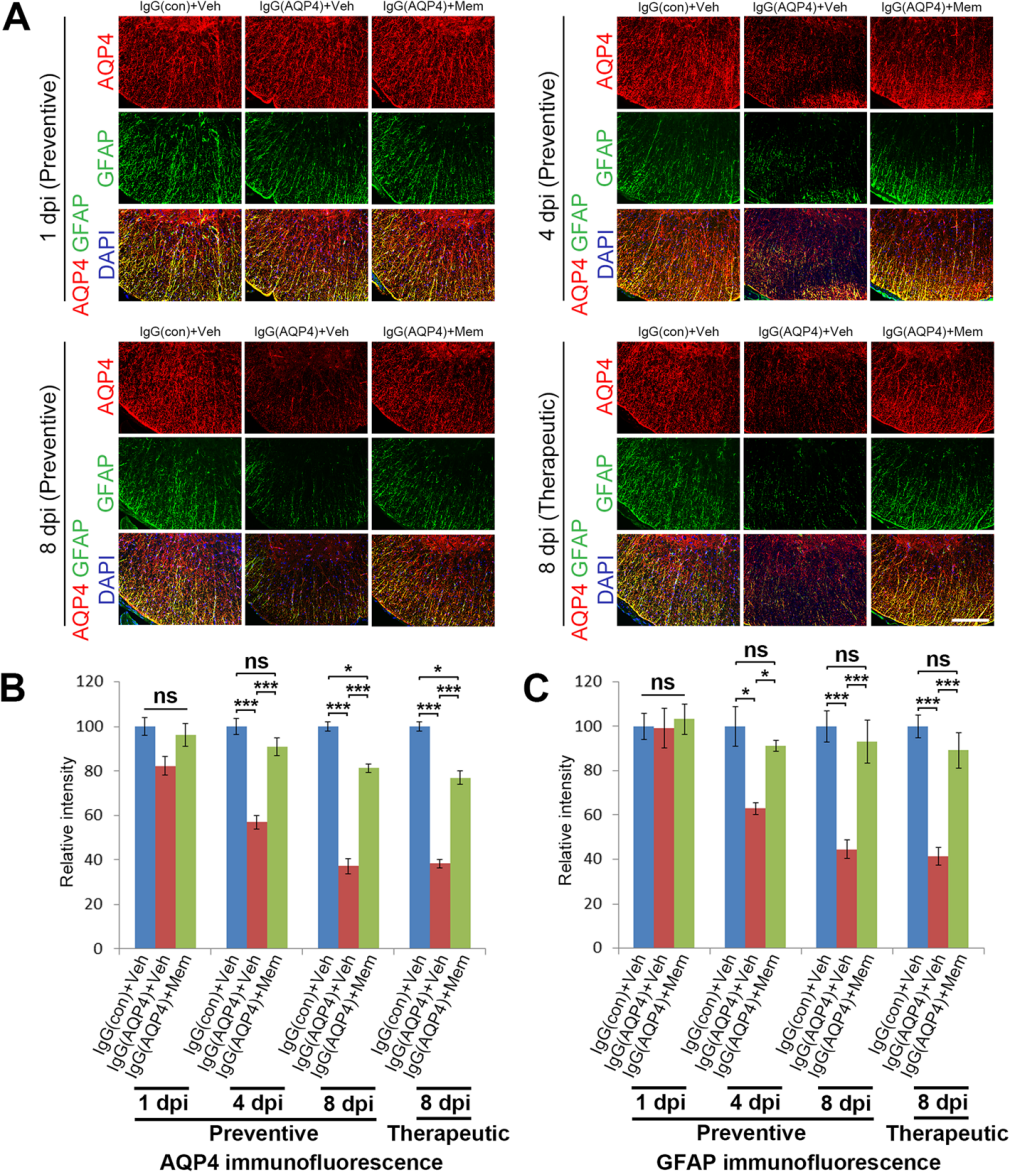
Memantine decreases AQP4 and GFAP loss in IgG(AQP4+) mice. Pictures are representative photomicrographs of cross sections showing the white matter in ventrolateral region of cervical spinal cord. **a** Double immunofluorescence staining of AQP4 and GFAP (astrocyte marker) in IgG(AQP4+) mice treated with preventive memantine or vehicle at 1, 4, and 8 dpi or with therapeutic memantine or vehicle at 8 dpi. IgG(con) mice treated with vehicle were used as a sham control. Sections were counterstained with DAPI. Scale bar = 100 μm. **b** Relative intensity of AQP4 immunofluorescence in the spinal cord. **c** Relative intensity of GFAP immunofluorescence in the spinal cord. *n* = 5 per group. Data are mean ± SEM. One-way ANOVA with Tukey-Kramer post hoc test. ns, not significant; *p < 0.05; ***p < 0.001

Our previous study has shown that passive transfer of human AQP4-IgG to mice with disrupted BBB does not cause complement activation in the spinal cord [13], consistent with the findings from others [21]. To evaluate the effects of memantine on AQP4-IgG-induced astrocytopathy, immunofluorescence analyses were performed. With pretreatment of CFA and PTx to disrupt the BBB, we found human IgG infiltration in the spinal cord parenchyma in all groups of mice (Additional file 2a, b). Moreover, we observed profound infiltration of human IgG in the area postrema, where there is a lack of BBB, as well as in the optic nerves (Additional file 2c, d). Double immunofluorescence staining revealed colocalization of AQP4 and GFAP in the spinal cord white matter of the mice. At 1 dpi, no differences on AQP4 and GFAP immunofluorescences in the spinal cords were observed between sham, vehicle-treated and memantine-treated IgG(AQP4+) mice. Loss of AQP4 and GFAP immunofluorescences began at 4 dpi in vehicle-treated IgG(AQP4+) mice. Both preventive and therapeutic treatment with memantine decreased the loss of AQP4 and GFAP immunofluorescences at 4 and 8 dpi compared to vehicle-treated IgG(AQP4+) mice (Fig. 2a). Evaluation of immunofluorescence intensity confirmed that the increases in AQP4 and GFAP levels in memantine-treated mice at 4 and 8 dpi compared to vehicle-treated IgG(AQP4+) mice were statistically significant (Fig. 2b, c). Similar findings were observed in the area postrema of the brain stem and the optic nerve (Additional file 5).

### Memantine ameliorated demyelination and axonal loss

**Fig. 3:**
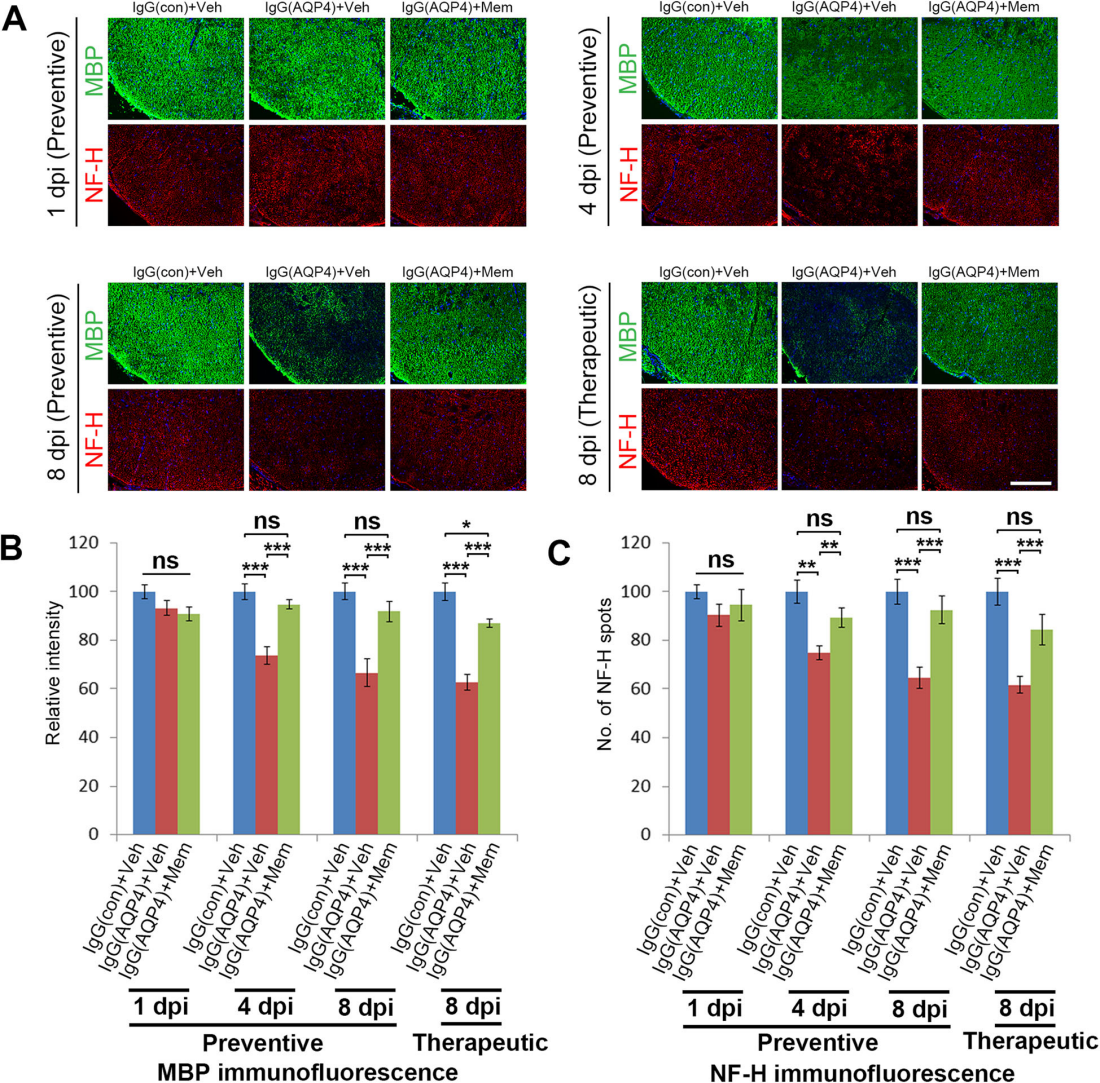
Memantine reduces demyelination and axonal loss in IgG(AQP4+) mice. **a** Immunofluorescence staining of MBP (myelin marker) and NF-H (axon marker) in the spinal cords of IgG(AQP4+) mice treated with preventive memantine or vehicle at 1, 4, and 8 dpi; or with therapeutic memantine or vehicle at 8 dpi. IgG(con) mice treated with vehicle were used as a sham control. Sections were counterstained with DAPI. Scale bar = 100 μm. **b** Relative intensity of MBP immunofluorescence in the spinal cord. **c** Quantitative analysis of the number of NF-H spots in the spinal cord normalized to sham. *n* = 5 per group. Data are mean ± SEM. One-way ANOVA with Tukey-Kramer post hoc test. ns, not significant; *p < 0.05; **p < 0.01; ***p < 0.001

We next examined whether memantine preserved myelin integrity and prevented axonal loss secondary to AQP4-IgG binding to astrocytes. MBP and NF-H immunofluorescence revealed, beginning from 4 dpi, prominent and patchy loss of myelin and axons in the spinal cord white matter of vehicle-treated IgG(AQP4+) mice compared to sham. Preventive and therapeutic treatment with memantine greatly increased MBP and NF-H signals at 4 and 8 dpi compared to vehicle-treated IgG(AQP4+) mice (Fig. 3a). Evaluation of immunofluorescence intensity confirmed that the increases in MBP level in memantine-treated mice at 4 and 8 dpi were statistically significant compared to vehicle-treated IgG(AQP4+) mice (Fig. 3b). Counting of NF-H-positive spots confirmed that at 4 and 8 dpi memantine-treated mice had more axons in the spinal cord than vehicle-treated IgG(AQP4+) mice (Fig. 3c). Luxol fast blue staining further showed that memantine preserved myelin integrity in the spinal cord of IgG(AQP4+) mice (Additional file 3). Similar findings were observed in the area postrema of the brain stem and the optic nerve (Additional file 6). These results indicated a protective effect of memantine against human AQP4-IgG-triggered demyelination and axonal loss in the mouse CNS.

### Memantine reduced NR2B expression and prevented apoptosis of astrocytes

**Fig. 4:**
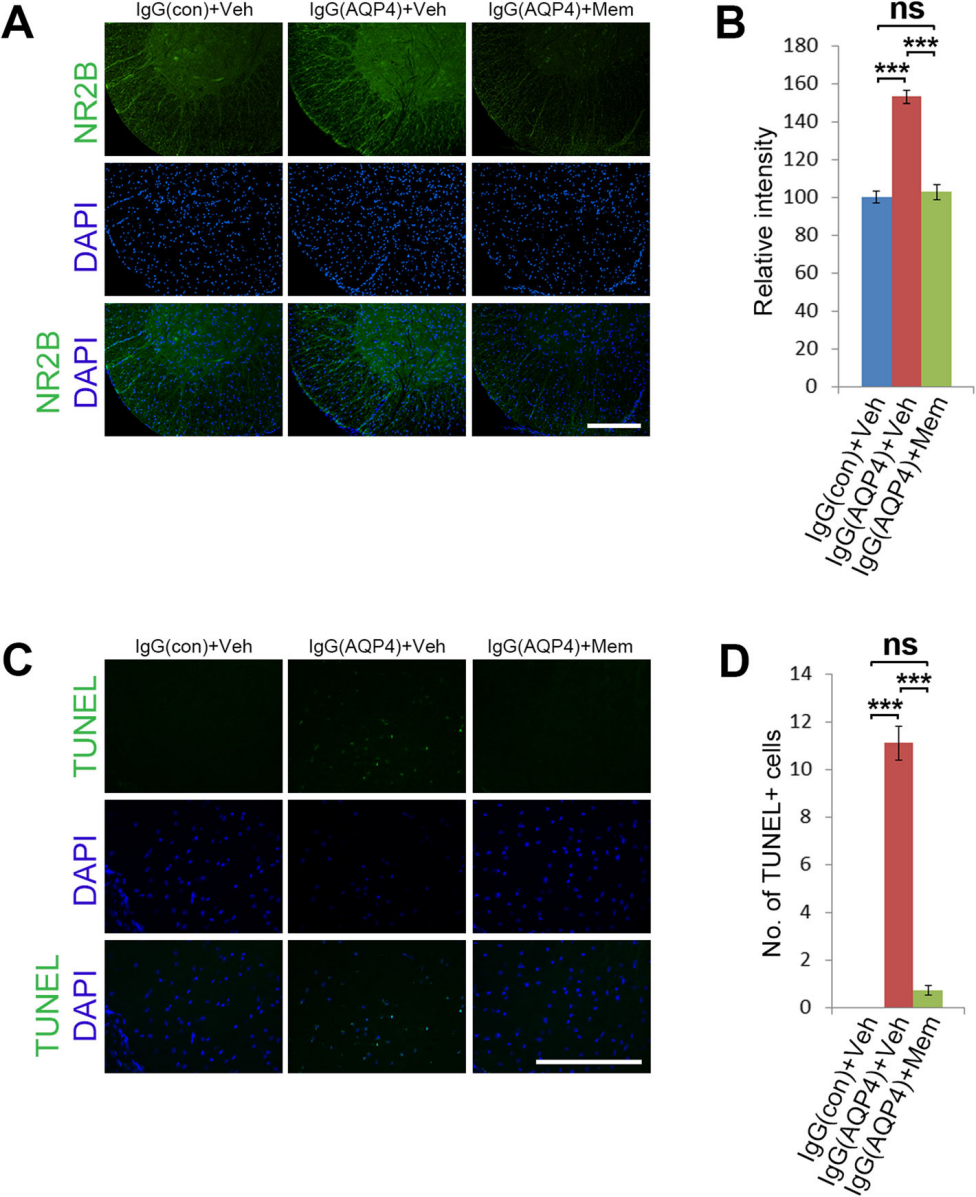
Memantine reduces NR2B expression and inhibits apoptosis in IgG(AQP4+) mice. **a** Immunofluorescence staining of NR2B in the spinal cords of IgG(AQP4+) mice treated with memantine or vehicle at 1 dpi. IgG(con) mice treated with vehicle were used as a sham control. Sections were counterstained with DAPI. Scale bar = 100 μm. **b** Relative intensity of NR2B immunofluorescence in the spinal cord. *n* = 5 per group. Data are mean ± SEM. One-way ANOVA with Tukey-Kramer post hoc test. ns, not significant; ***p < 0.001. **c** TUNEL staining of apoptotic cells in the spinal cords of IgG(AQP4+) mice treated with memantine or vehicle at 1 dpi. IgG(con) mice treated with vehicle were used as a sham control. Sections were counterstained with DAPI. Scale bar = 100 μm. **d** Quantitative analysis of the number of TUNEL-positive cells in the spinal cord. *n* = 5 per group. Data are mean ± SEM. One-way ANOVA with Tukey-Kramer post hoc test. ns, not significant; ***p < 0.001

Memantine has been shown to downregulate the NMDA receptor subunit NR2B, which is predominantly localized to extrasynaptic NMDA receptors [22–24]. To examine whether memantine decreased NR2B expression in our model, immunofluorescence analysis was performed. At 1 dpi, the level of NR2B expression in the spinal cord white matter of vehicle-treated IgG(AQP4+) mice were higher than that in sham mice. Memantine treatment profoundly reduced the NR2B expression in the spinal cord of IgG(AQP4+) mice to sham level (Fig. 4a). Evaluation of immunofluorescence intensity confirmed that the reduction in NR2B level in memantine-treated mice compared to vehicle-treated IgG(AQP4+) mice was statistically significant (Fig. 4b).

To investigate whether memantine prevented cell loss by apoptosis, we detected DNA fragmentation using the TUNEL assay. At 1 dpi, apoptotic cells in the spinal cord white matter of vehicle-treated IgG(AQP4+) mice were more than that in sham mice. Memantine treatment markedly reduced apoptotic cells (Fig. 4c). Quantitative analysis revealed that the number of TUNEL-positive cells was significantly reduced in the memantine-treated mice, compared to the vehicle-treated IgG(AQP4+) mice (Fig. 4d). At 4 and 8 dpi, no apoptotic cells were found in the spinal cord of mice in all groups (data not shown). These results suggested that astrocytic apoptosis is an early event upon AQP4-IgG binding to astrocytes, and memantine treatment could prevent apoptotic cell death of astrocytes induced by human AQP4-IgG in mouse spinal cord.

### Memantine attenuated microglia activation and neutrophil infiltration

**Fig. 5:**
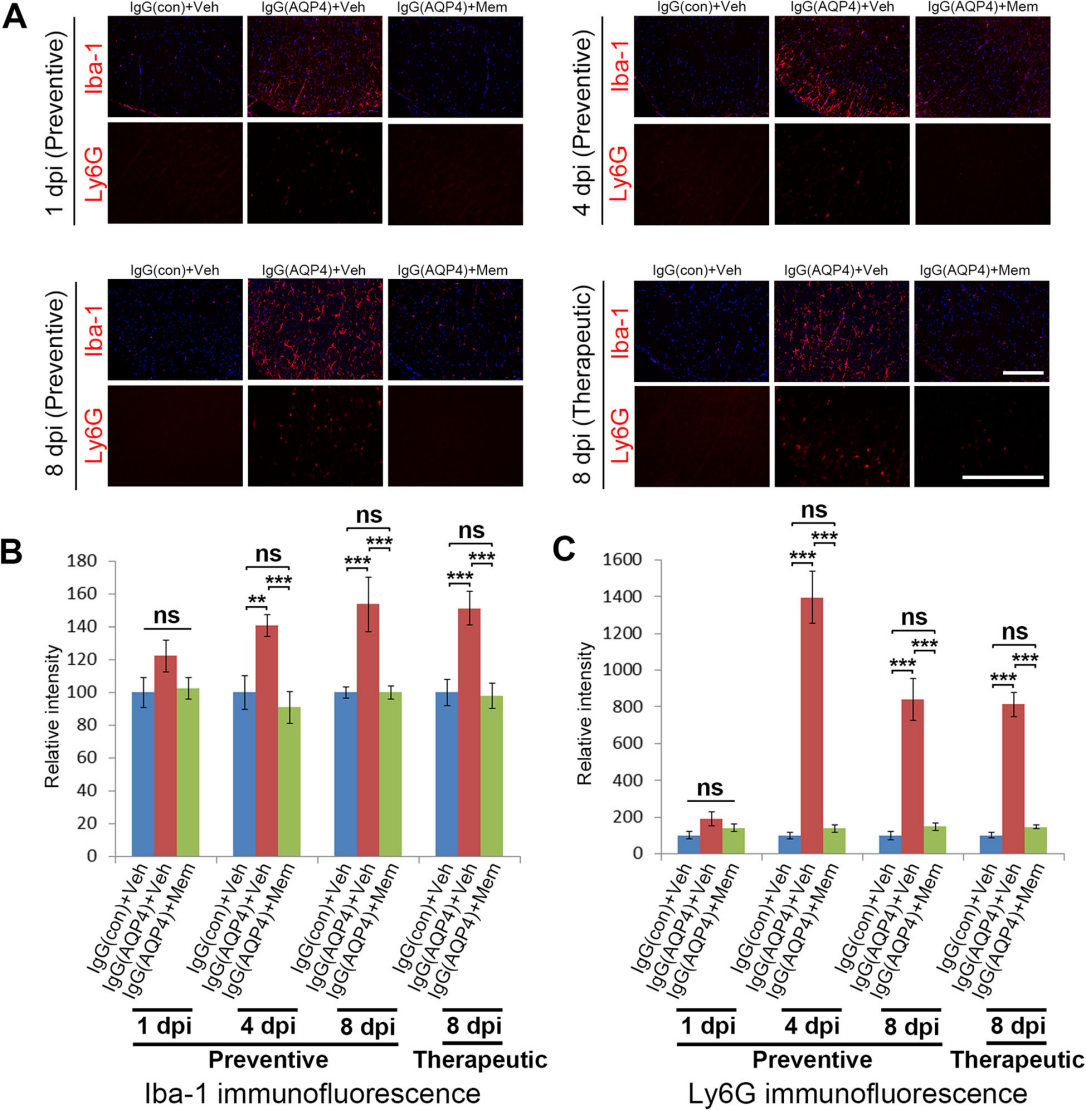
Memantine suppresses microglia activation and neutrophil infiltration. **a** Immunofluorescence staining of Iba-1 (microglia marker) and Ly6G (neutrophil marker) in the spinal cords of IgG(AQP4+) mice treated with preventive memantine or vehicle at 1, 4, and 8 dpi or with therapeutic memantine or vehicle at 8 dpi. IgG(con) mice treated with vehicle were used as a sham control. Sections were counterstained with DAPI. Scale bar = 100 μm. **b** Relative intensity of Iba-1 immunofluorescence in the spinal cord. **c** Relative intensity of Ly6G immunofluorescence in the spinal cord. *n* = 5 per group. Data are mean ± SEM. One-way ANOVA with Tukey-Kramer post hoc test. ns, not significant; **p < 0.01; ***p < 0.001

To examine whether memantine influenced neuroinflammation triggered by human AQP4-IgG, immunofluorescence analyses on microglia activation and neutrophil infiltration were performed. At 1 dpi, no differences on microglia activation, as evaluated by Iba-1 immunofluorescence, and neutrophil infiltration, as evaluated by Ly6G immunofluorescence, were observed between the spinal cords of sham, vehicle-treated, and memantine-treated IgG(AQP4+) mice. Profound increases in Iba-1 and Ly6G immunofluorescences were observed since 4 dpi in vehicle-treated IgG(AQP4+) mice. At 4 and 8 dpi, both preventive and therapeutic treatment with memantine decreased Iba-1 and Ly6G immunofluorescences compared to vehicle-treated IgG(AQP4+) mice (Fig. 5a). Evaluation of immunofluorescence intensity confirmed that the decreases in Iba-1 and Ly6G levels in memantine-treated mice at 4 and 8 dpi compared to vehicle-treated IgG(AQP4+) mice were statistically significant (Fig. 5b, c). H&E staining revealed tissue damage and inflammatory cell infiltration in the spinal cord parenchyma of vehicle-treated IgG(AQP4+) mice, while these were markedly reduced with memantine treatments (Additional file 3). These findings indicated that memantine suppressed AQP4-IgG-induced microglia activation and neutrophil infiltration in the spinal cord. To further examine if T cells infiltrated into the spinal cord in our model, immunofluorescence staining of CD4 (T helper cells) and CD8 (T cytotoxic cells) were performed. No T cell infiltration was observed in the spinal cords of sham, vehicle-treated, and memantine-treated IgG(AQP4+) mice (Additional file 4). These findings suggested that the beneficial effects of memantine did not involve modulation of T cell infiltration into the spinal cord.

### Memantine reduced concentrations of proinflammatory cytokines in the spinal cord

**Fig. 6:**
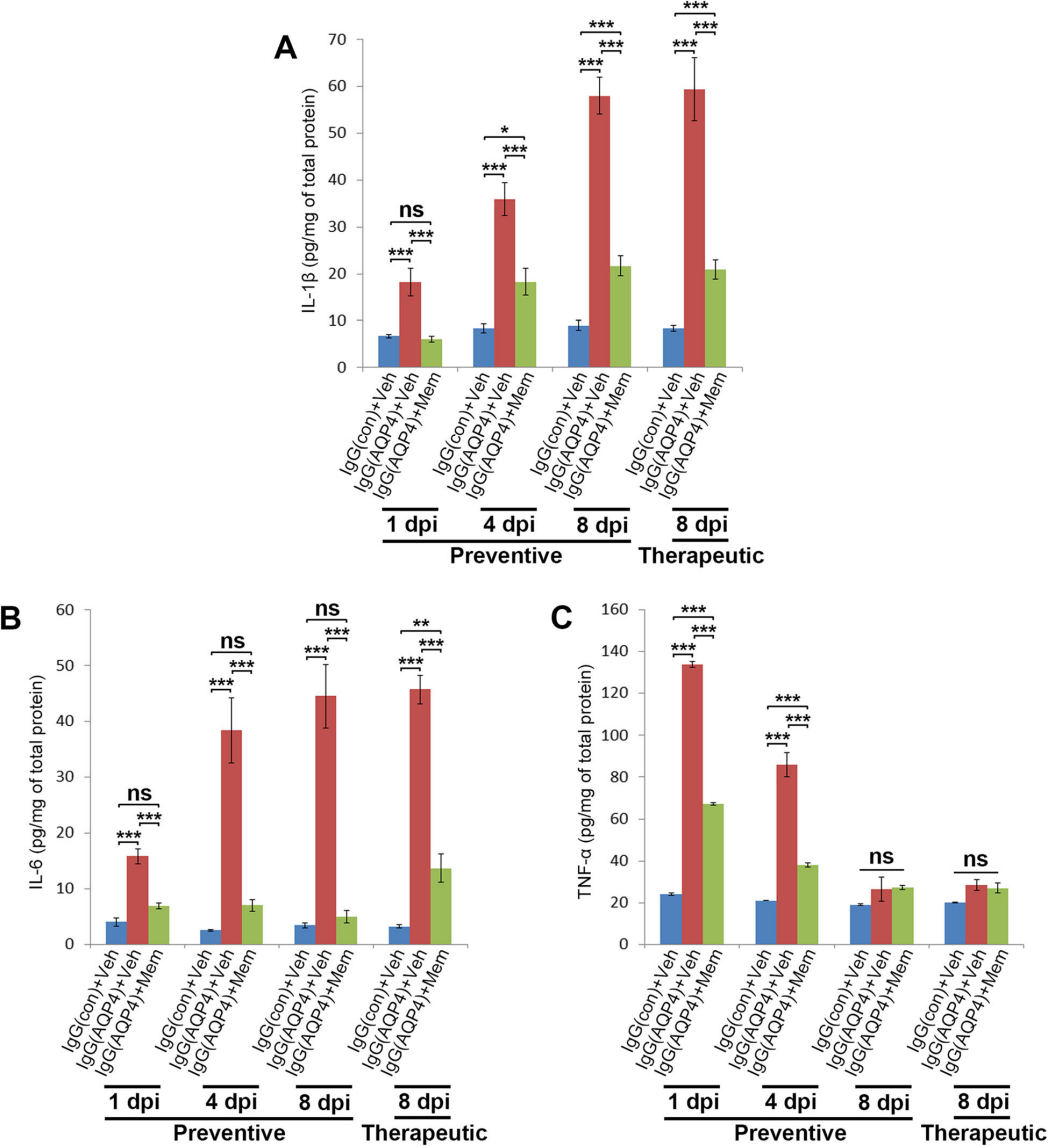
Reduction in proinflammatory cytokines in memantine-treated IgG(AQP4+) mice. **a**–**c** ELISA analyses of IL-1β (**a**), IL-6 (**b**), and TNF-α (**c**) in the spinal cords of IgG(AQP4+) mice treated with preventive memantine or vehicle at 1, 4, and 8 dpi or with therapeutic memantine or vehicle at 8 dpi. IgG(con) mice treated with vehicle were used as a sham control. *n* = 3 per group. Each ELISA was performed in duplicates. Data are mean ± SEM. One-way ANOVA with Tukey-Kramer post hoc test. ns, not significant; *p < 0.05; **p < 0.01; ***p < 0.001

Activated microglia releases proinflammatory cytokines such as IL-1β, IL-6, and TNF-α [25]. AQP4-IgG can induce astrocytes to release a broad spectrum of proinflammatory mediators including IL-1β and IL-6 [11]. To investigate whether memantine treatment reduced neuroinflammation, ELISA of IL-1β, IL-6 and TNF-α were performed in spinal cord homogenates of mice in different groups. Results revealed that concentrations of IL-1β and IL-6 in the spinal cords of vehicle-treated IgG(AQP4+) mice were markedly higher than that of sham mice at all the time points examined. Both preventive and therapeutic treatment with memantine significantly reduced IL-1β and IL-6 concentrations compared to vehicle-treated IgG(AQP4+) mice (Fig. 6a, b). The concentration of TNF-α in the spinal cords of vehicle-treated IgG(AQP4+) mice was markedly higher than that of sham mice at 1 and 4 dpi, and then it decreased to that similar to sham. Preventive treatment with memantine significantly reduced TNF-α concentration at 1 and 4 dpi compared to vehicle-treated IgG(AQP4+) mice (Fig. 6c).

### Memantine elevated concentrations of growth factors in the spinal cord

**Fig. 7:**
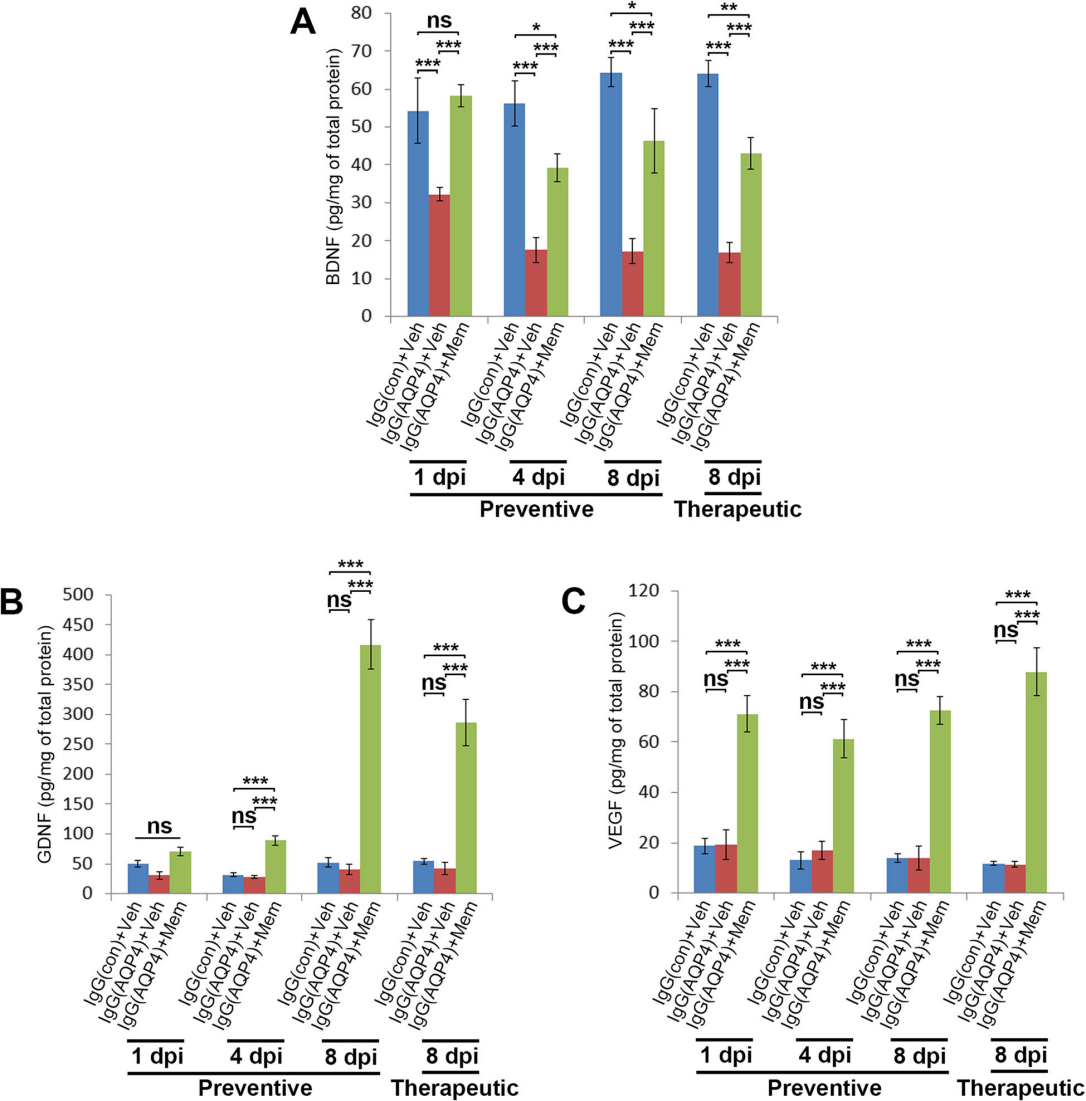
Elevation of growth factors in memantine-treated IgG(AQP4+) mice. **a**–**c** ELISA analyses of BDNF (**a**), GDNF (**b**), and VEGF (**c**) in the spinal cords of IgG(AQP4+) mice treated with preventive memantine or vehicle at 1, 4, and 8 dpi or with therapeutic memantine or vehicle at 8 dpi. IgG(con) mice treated with vehicle were used as a sham control. *n* = 3 per group. Each ELISA was performed in duplicates. Data are mean ± SEM. One-way ANOVA with Tukey-Kramer post hoc test. ns, not significant; *p < 0.05; **p < 0.01; ***p < 0.001

To further characterize the protective effects of memantine, we performed ELISA to evaluate the concentrations of BDNF, GDNF, and VEGF in spinal cord homogenates of mice in different groups. Results revealed that BDNF concentration in the spinal cords of vehicle-treated IgG(AQP4+) mice was markedly lower than that of sham mice at all the time points examined. Both preventive and therapeutic treatment with memantine significantly increased BDNF concentration compared to vehicle-treated IgG(AQP4+) mice (Fig. 7a). GDNF concentration in the spinal cord of vehicle-treated IgG(AQP4+) mice did not differ significantly compared to sham mice at all the time points examined. Both preventive and therapeutic treatment with memantine significantly increased GDNF concentration compared to sham- and vehicle-treated IgG(AQP4+) mice at 4 and 8 dpi (Fig. 7b). Similarly, VEGF concentration in the spinal cord of vehicle-treated IgG(AQP4+) mice did not differ significantly compared to sham mice. The memantine treatments significantly increased VEGF concentration compared to sham and vehicle-treated IgG(AQP4+) mice at all the time points examined (Fig. 7c).

## Discussion

We recently reported that passive transfer of human AQP4-IgG to mice with disrupted BBB led to complement-independent NMOSD-like spinal cord pathologies of AQP4 and astrocyte loss, EAAT2 loss, microglia activation, neutrophil infiltration, demyelination, and axonal loss, which were associated with motor impairments [13]. Here, we showed that memantine, when administered either simultaneously with or 4 days after passive transfer of human AQP4-IgG, ameliorated motor impairments in mice associated with marked alleviation of NMOSD-like pathologies, including decrease of AQP4 and GFAP loss, and reduction of demyelination and axonal loss. The mechanisms underlying the preventive and therapeutic effects of memantine involved prevention of apoptotic cell death, suppression of microglia activation and neutrophil infiltration, reduction of proinflammatory cytokines, and elevation of growth factors. These results indicate important pathological roles of glutamate excitotoxicity and neuroinflammation in NMOSD acute attacks, and highlight the potential of memantine as a therapeutic agent in NMOSD acute attacks.

We found memantine markedly prevented and ameliorated astrocyte loss, demyelination and axonal loss in IgG(AQP4+) mice. The primary mechanism of memantine’s beneficial effects may be attributed to the reduction of glutamate excitotoxicity against astrocytes, oligodendrocytes and neurons. Interestingly, memantine could not completely reverse the AQP4 loss (up to 70-80% as shown in Figure 2b), suggesting that the upstream immunological activity, including AQP4 loss due to internalization of autoantigen-autoantibody complexes, persisted after memantine treatment. Our previous study found that AQP4-IgG caused reduction of glutamate transporter EAAT2, suggesting a decrease in glutamate uptake [13]. Memantine has been shown to downregulate NR2B in different models of CNS insults [22–24]. In this study, we found that AQP4-IgG induced upregulation of NR2B expression, which was reversed by memantine treatment. The protective effects of memantine may be attributed to its inhibitory action on extrasynaptic glutamate receptors, which may stimulate cell death pathways in glia and neurons. Moreover, astrocyte is an important component of the BBB. Prevention of glutamate excitotoxicity-induced astrocyte loss by memantine may help to preserve BBB integrity.

Memantine is approved for the treatment of Alzheimer’s disease. It is an open-channel blocker of NMDA receptor with faster blocking/unblocking kinetics and stronger voltage dependency than other competitive and non-competitive NMDA receptor antagonists [26]. These properties account for the better safety and tolerability of memantine compared to other NMDA receptor blockers, such as MK-801 and phencyclidine, which may cause negative psychotropic effects [26, 27]. Memantine is an effective blocker of NMDA receptor only during pathological conditions, without affecting normal physiological functions. We found that both preventive and therapeutic treatment with memantine improved motor performance in mice which had received human AQP4-IgG. To our knowledge, this is the first study to show a clinical benefit of memantine in a NMOSD model.

Following AQP4-IgG binding to astrocytic AQP4, complement-independent pathophysiologies have been suggested to be important early processes in the development of NMOSD lesions [10, 28]. AQP4-IgG binding causes internalization of AQP4 together with the glutamate transporter EAAT2 from astrocytic membrane. This reduces the uptake of extracellular glutamate by astrocytes and disrupts glutamate homeostasis, leading to glutamate excitotoxicity against NMDA receptor-expressing glial cells and neurons [9, 29–32]. A recent study demonstrated that co-endocytosis of AQP4 and EAAT2 on astrocytic membrane upon AQP4-IgG binding required astrocytic Fcγ receptor, supporting a role of glutamate excitotoxicity in the early pathophysiologies of NMOSD acute attacks [10]. AQP4-IgG from NMOSD patients caused astrocyte injury with secondary damage to oligodendrocytes mediated by glutamate excitotoxicity, which may contribute to the demyelination observed in our model [32]. Overstimulation of NMDA receptors by glutamate triggers excessive Ca^2+^ influx, mitochondrial dysfunction, oxidative stress, and apoptotic cell death [33]. We found that memantine prevented astrocyte loss, demyelination, and axonal loss in mice which had received human AQP4-IgG. We also detected significantly more apoptotic cells in the spinal cord white matter of vehicle-treated mice than that of memantine-treated mice early (1 day) after passive transfer of AQP4-IgG. Our previous study found that human AQP4-IgG did not cause significant loss of oligodendrocytes and neurons in this mouse model [13]. The apoptotic cells in the white matter observed in the current study were likely astrocytes. These findings suggested that memantine protected astrocytes from glutamate excitotoxicity and apoptosis that were triggered by AQP4-IgG binding. Indeed, loss of EAAT2 was observed in the spinal cord lesion of NMOSD patients, supporting a role of glutamate exicitotoxicity in NMOSD pathophysiologies in human [5].

AQP4-IgG binding stimulates astrocytes to release proinflammatory cytokines, chemokines, and other inflammatory mediators that can activate microglia, thus promote further production of inflammatory mediators in a vicious cycle [11]. This aggravates neuroinflammation which involves microglia activation and parenchymal infiltration of macrophages, granulocytes, and natural killer cells from the peripheral circulation. These activated immune cells with Fc receptors can lead to astrocyte cytotoxicity via ADCC [12, 34, 35]. The protective effect of memantine on astrocytes likely attenuated the release of proinflammatory cytokines and chemokines from astrocytes triggered by AQP4-IgG binding, hence reducing microglia activation. In the EAE model of multiple sclerosis, memantine reduced neurological symptoms and decreased the expression of proinflammatory cytokines in the brain [36]. In the mouse cuprizone model of demyelination, memantine inhibits the production of proinflammatory cytokines by astrocytes via modulation of NMDA receptor [37]. Moreover, the anti-inflammatory property of memantine was shown to be mediated through inhibition of microglia activation [38]. Consistent with these studies, we found that memantine suppressed human AQP4-IgG-triggered microglia activation and neutrophil infiltration, and reduced proinflammatory IL-1β, IL-6, and TNF-α levels in mouse spinal cord. These findings suggested that memantine could exert anti-inflammatory effects through cellular mechanisms secondary to blocking NMDA receptors.

Neurotrophic factors play a critical role in the maintenance and survival of glial cells and neurons in the adult CNS. Astrocytes have been found to be a major source of neurotrophic factors [39]. Memantine markedly increased the levels of BDNF and its receptor trkB across the brain [40]. In dopaminergic neuron-glia cocultures, the neuroprotective effects of memantine was mediated by stimulating GDNF release from astrocytes [38]. In a streptozotocin-induced astrocytotoxicity model for Alzheimer’s disease, memantine ameliorated BDNF and GDNF decline in astrocytes along with phosphorylation of IRS-1, Akt, and GSK-3α/β [41]. On the other hand, astrocytes were shown to produce VEGF upon brain injury, ischemia, and neuroinflammation for regulating vascular remodeling and angiogenesis during the repair process [42, 43]. Memantine improved stroke outcomes via increasing BDNF, GDNF, and VEGF levels, reducing reactive astrogliosis and enhancing vascular density [24, 44]. In a rat model of chronic cerebral hypoperfusion, memantine enhanced neovascularization with increases in BDNF and VEGF expressions [45]. The precise mechanism of how memantine enhances the production of growth factors is not clear. Interestingly, in addition to its antagonistic effect on NMDA receptors, memantine is a highly lipophilic agent that can readily enter cells and act on intracellular processes [27]. We found that memantine prevented astrocyte loss and simultaneously increased BDNF, GDNF, and VEGF levels in the spinal cord of mice which had received human AQP4-IgG. These results suggested that the elevated growth factor levels likely associated with the protective effect of memantine on astrocytes.

A limitation of our study is that complement-dependent pathophysiologies were absent in our model. In addition, the dose of memantine used in this study was higher than that in human patients with Alzheimer’s disease (maintenance dose: 20 mg orally per day). The serum half-life of memantine in rodents is shorter than that in human [46]. Memantine will likely exert neuroprotective effects on patients with NMOSD at commonly used doses such as that for Alzheimer’s disease. Another limitation of this study is that we did not examine if memantine blocks NMDA-R in our model.

## Conclusions

In summary, we found that memantine markedly ameliorated complement-independent motor impairments and spinal cord pathologies induced by human AQP4-IgG in mice. These findings suggested important roles of glutamate excitotoxicity and neuroinflammation in early pathophysiologies of NMOSD attacks. Memantine is a well-tolerated oral drug. Our results support perspectives for clinical studies on the use of memantine as preventive and therapeutic treatments of NMOSD acute attacks.

## Supplementary information

**Additional file 1.** Preventive memantine at 60 mg/kg/day, but not 20 mg/kg/day ameliorates human AQP4-IgG-induced motor impairments in mice. Mice were pretreated with CFA and PTx. From day 0, IgG(AQP4+) mice received daily i.p. injection of purified IgG from AQP4-IgG-seropositive NMOSD patients and oral gavage of vehicle, memantine at 20 mg/kg/day (Mem 20) or 60 mg/kg/day (Mem 60) till 7 dpi. Beam walking test was performed at 8 dpi. a-b Time required a and number of paw slips b during walking across a 1.2 x 80 cm (width x length) beam in beam walking test on IgG(AQP4+) mice treated with vehicle, Mem 20 or Mem 60 at 8 dpi. IgG(con) mice treated with vehicle were used as a sham control. c-d Time required c and number of paw slips d during walking across a 0.6 x 80 cm (width x length) beam in beam walking test on mice in different groups. n = 8 per group. Data are mean ± SEM. One-way ANOVA with Tukey-Kramer post hoc test. ns, not significant; *p < 0.05; ***p < 0.001. e H&E staining of longitudinal sections of hind limb muscle of IgG(con) mice, and IgG(AQP4+) mice treated with vehicle or Mem 60 at 8 dpi. Scale bars = 100 μm.

**Additional file 2.** Infiltration of human IgG in different animal groups. a Representative photomicrographs of cross sections showing the white matter in ventrolateral region of cervical spinal cord. Immunofluorescence staining of human IgG in the spinal cords of IgG(AQP4+) mice treated with preventive memantine or vehicle at 1, 4, 8 dpi; or with therapeutic memantine or vehicle at 8 dpi. IgG(con) mice treated with vehicle were used as a sham control. Sections were counterstained with DAPI. b Lower magnification photomicrographs of spinal cord cross sections showing human IgG immunofluorescence. c-d Representative photomicrographs of brain stem (area postrema) cross sections c and optic nerve longitudinal sections d showing human IgG immunofluorescence. Scale bars = 100 μm.

**Additional file 3.** Histopathology of spinal cords of mice in different groups. Pictures are representative photomicrographs of cross sections showing the white matter in ventrolateral region of cervical spinal cord. Luxol fast blue and H&E staining of the spinal cords of IgG(AQP4+) mice treated with preventive memantine or vehicle at 1, 4, 8 dpi; or with therapeutic memantine or vehicle at 8 dpi. IgG(con) mice treated with vehicle were used as a sham control. Scale bar = 100 μm.

**Additional file 4.** No T cells infiltration to the spinal cord. a Immunofluorescence staining of CD4 (T helper cell marker) and CD8 (T cytotoxic cell marker) in the spinal cords of IgG(AQP4+) mice treated with therapeutic memantine or vehicle at 8 dpi. IgG(con) mice treated with vehicle were used as a sham control. Spleens were used as a positive staining control. Sections were counterstained with DAPI. Photomicrographs are representatives of 5 animals from each group. Scale bar = 100 μm.

**Additional file 5.** Memantine decreases AQP4 and GFAP loss in the area postrema and optic nerves of IgG(AQP4+) mice. a Representative photomicrographs of brain stem cross sections showing the area postrema. Double immunofluorescence staining of AQP4 and GFAP in IgG(AQP4+) mice treated with therapeutic memantine or vehicle at 8 dpi. IgG(con) mice treated with vehicle were used as a sham control. b Relative intensity of AQP4 and GFAP immunofluorescence in the area postrema. c Representative photomicrographs of longitudinal sections of the optic nerve region proximal to the optic chiasm. Double immunofluorescence staining of AQP4 and GFAP in IgG(AQP4+) mice treated with therapeutic memantine or vehicle at 8 dpi. IgG(con) mice treated with vehicle were used as a sham control. d Relative intensity of AQP4 and GFAP immunofluorescence in the optic nerve. Dotted lines demarcate the areas where quantifications of fluorescence intensities were performed. n = 3 per group. Data are mean ± SEM. One-way ANOVA with Tukey-Kramer post hoc test. ns, not significant; ***p < 0.001. Scale bar = 100 μm.

**Additional file 6.** Memantine reduces demyelination and axonal loss in the area postrema and optic nerves of IgG(AQP4+) mice. a Representative photomicrographs of brain stem cross sections showing the area postrema. Double immunofluorescence staining of MBP and NF-H in IgG(AQP4+) mice treated with therapeutic memantine or vehicle at 8 dpi. IgG(con) mice treated with vehicle were used as a sham control. b Relative intensity of MBP and NF-H immunofluorescence in the area postrema. c Representative photomicrographs of longitudinal sections of the optic nerve region proximal to the optic chiasm. Double immunofluorescence staining of MBP and NF-H in IgG(AQP4+) mice treated with therapeutic memantine or vehicle at 8 dpi. IgG(con) mice treated with vehicle were used as a sham control. d Relative intensity of MBP and NF-H immunofluorescence in the optic nerve. n = 3 per group. Dotted lines demarcate the areas where quantifications of fluorescence intensities were performed. Data are mean ± SEM. One-way ANOVA with Tukey-Kramer post hoc test. ns, not significant; ***p < 0.001. Scale bar = 100 μm.

## Data Availability

The datasets used and/or analyzed during the current study are available from the corresponding author on reasonable request.

## Abbreviations

ADCC: Antibody-dependent cell-mediated cytotoxicity
ANOVA: Analysis of variance
AQP4: Aquaporin-4
AQP4-IgG: Aquaporin-4 immunoglobulin G
BDNF: Brain-derived neurotrophic factor
CFA: Complete Freund’s adjuvant
CNS: Central nervous system
EAAT2: Excitatory amino acid transporter 2
GDNF: Glial cell line-derived neurotrophic factor
GFAP: Glial fibrillary acidic protein
H&E: Hematoxylin and eosin
Iba-1: Ionized calcium-binding adapter molecule 1
IL-1β: Interleukin-1β
IL-6: Interleukin-6
LFB: Luxol fast blue
Ly6G: Lymphocyte antigen 6 complex locus G6D
MBP: Myelin basic protein
NF-H: Neurofilament heavy polypeptide
NMDA: N-methyl-D-aspartate
NMOSD: Neuromyelitis optica spectrum disorders
PTx: Pertussis toxin
TNF-α: Tumor necrosis factor-α
TUNEL: Terminal deoxynucleotidyl transferase dUTP nick-end labeling
VEGF: Vascular endothelial growth factor
