# Supplementary figures and images for: Memantine ameliorates motor impairments and pathologies in a mouse model of neuromyelitis optica spectrum disorders
Source: J Neuroinflammation. 2020 Aug 11;17:236. doi: 10.1186/s12974-020-01913-2 (PMC7418436; doi:10.1186/s12974-020-01913-2)

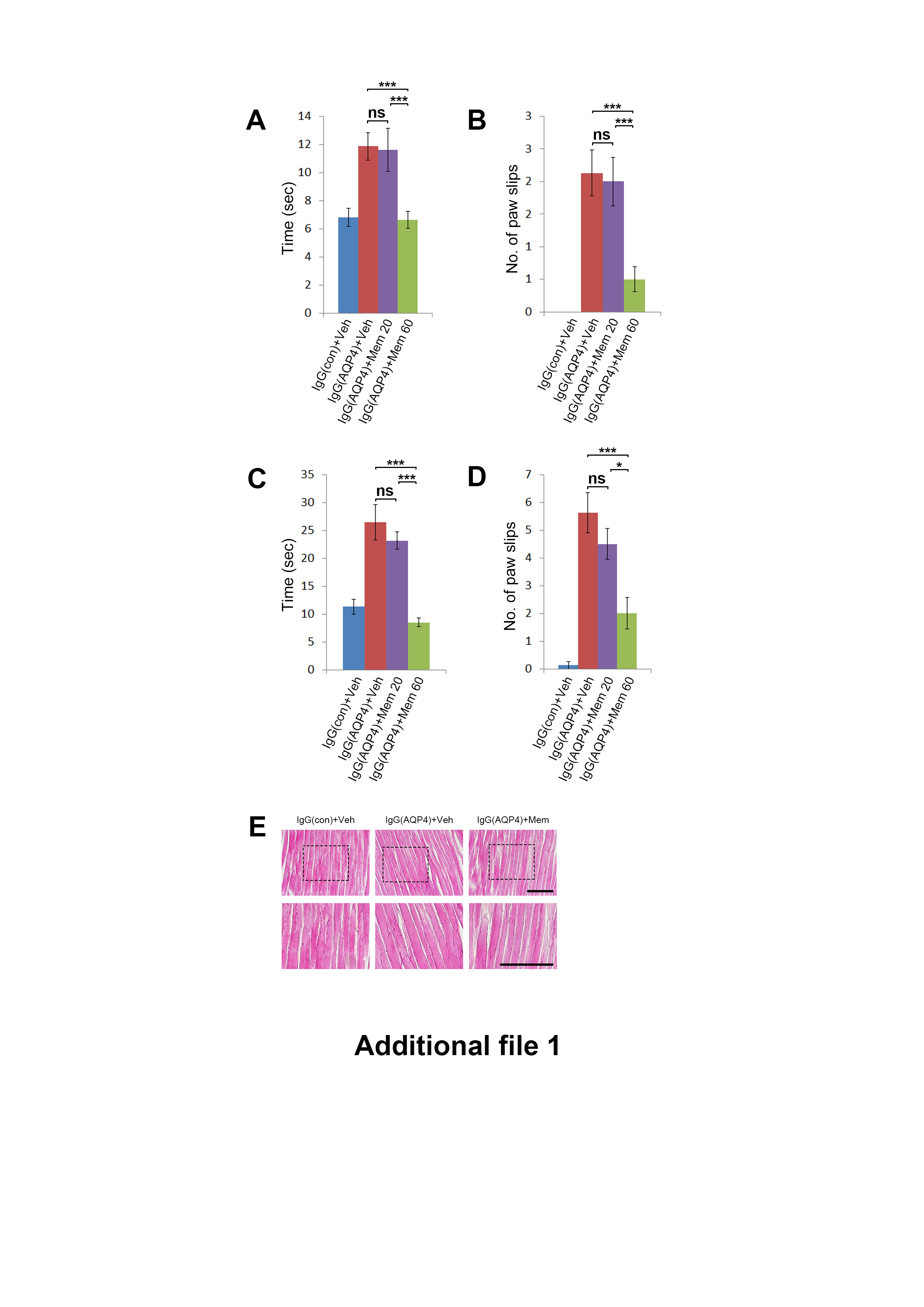

Supplement: Supplementary file 9 — Additional file 1. Preventive memantine at 60 mg/kg/day, but not 20 mg/kg/day ameliorates human AQP4-IgG-induced motor impairments in mice. Mice were pretreated with CFA and PTx. From day 0, IgG(AQP4+) mice received daily i.p. injection of purified IgG from AQP4-IgG-seropositive NMOSD patients and oral gavage of vehicle, memantine at 20 mg/kg/day (Mem 20) or 60 mg/kg/day (Mem 60) till 7 dpi. Beam walking test was performed at 8 dpi. a-b Time required a and number of paw slips b during walking across a 1.2 x 80 cm (width x length) beam in beam walking test on IgG(AQP4+) mice treated with vehicle, Mem 20 or Mem 60 at 8 dpi. IgG(con) mice treated with vehicle were used as a sham control. c-d Time required c and number of paw slips d during walking across a 0.6 x 80 cm (width x length) beam in beam walking test on mice in different groups. n = 8 per group. Data are mean ± SEM. One-way ANOVA with Tukey-Kramer post hoc test. ns, not significant; *p < 0.05; ***p < 0.001. e H&E staining of longitudinal sections of hind limb muscle of IgG(con) mice, and IgG(AQP4+) mice treated with vehicle or Mem 60 at 8 dpi. Scale bars = 100 μm. [file 12974_2020_1913_MOESM1_ESM.tif]

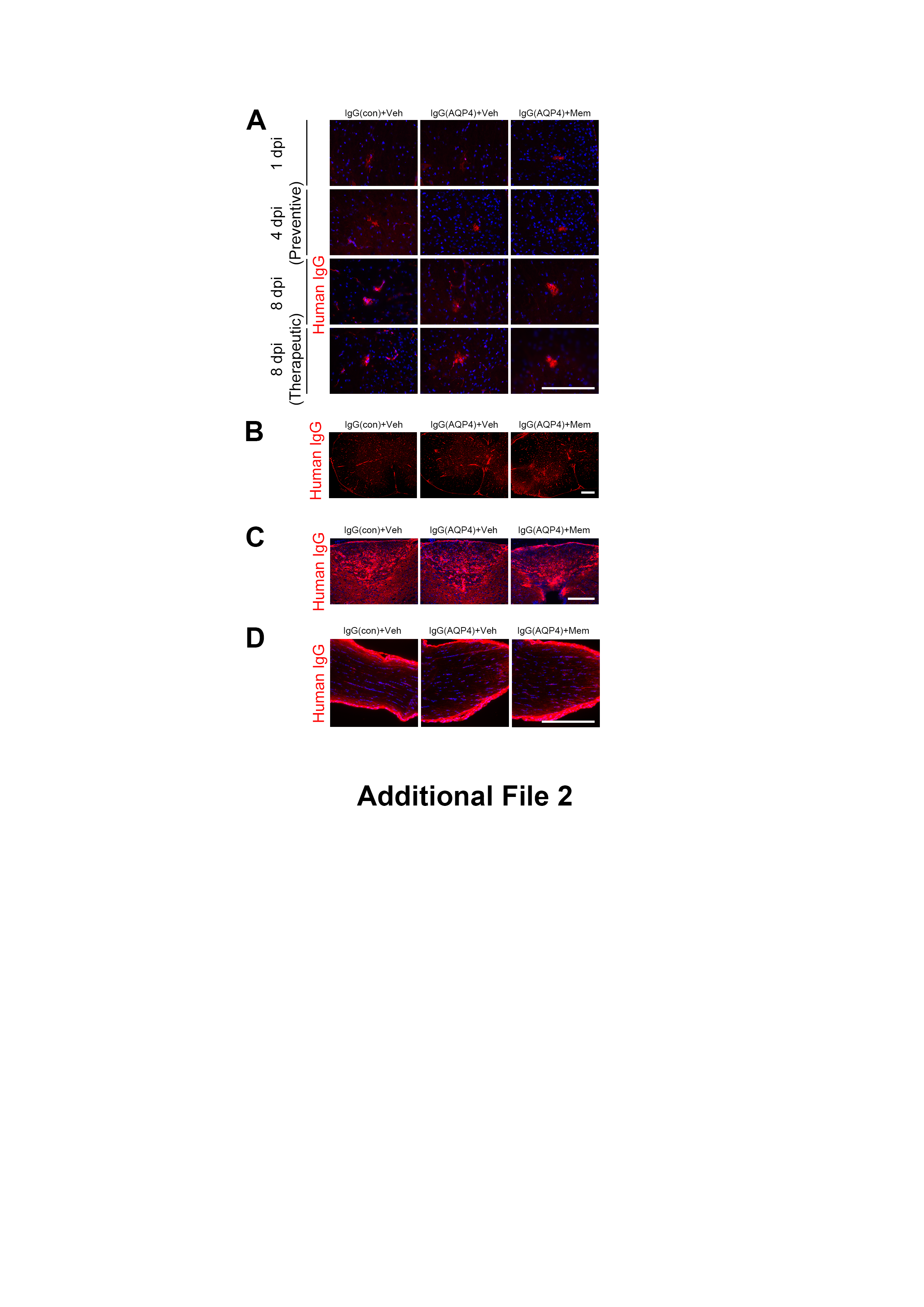

Supplement: Supplementary file 10 — Additional file 2. Infiltration of human IgG in different animal groups. a Representative photomicrographs of cross sections showing the white matter in ventrolateral region of cervical spinal cord. Immunofluorescence staining of human IgG in the spinal cords of IgG(AQP4+) mice treated with preventive memantine or vehicle at 1, 4, 8 dpi; or with therapeutic memantine or vehicle at 8 dpi. IgG(con) mice treated with vehicle were used as a sham control. Sections were counterstained with DAPI. b Lower magnification photomicrographs of spinal cord cross sections showing human IgG immunofluorescence. c-d Representative photomicrographs of brain stem (area postrema) cross sections c and optic nerve longitudinal sections d showing human IgG immunofluorescence. Scale bars = 100 μm. [file 12974_2020_1913_MOESM2_ESM.tif]

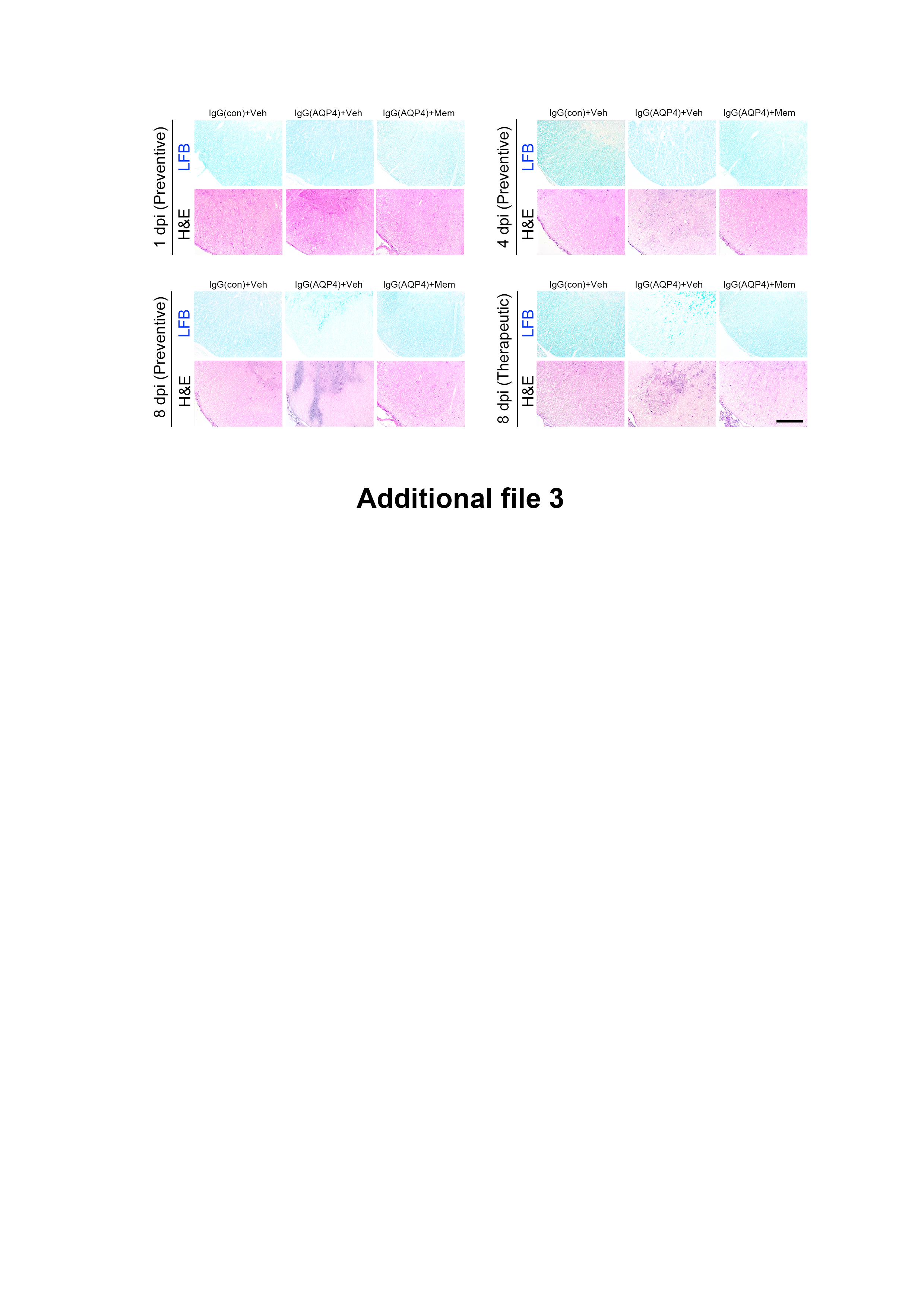

Supplement: Supplementary file 11 — Additional file 3. Histopathology of spinal cords of mice in different groups. Pictures are representative photomicrographs of cross sections showing the white matter in ventrolateral region of cervical spinal cord. Luxol fast blue and H&E staining of the spinal cords of IgG(AQP4+) mice treated with preventive memantine or vehicle at 1, 4, 8 dpi; or with therapeutic memantine or vehicle at 8 dpi. IgG(con) mice treated with vehicle were used as a sham control. Scale bar = 100 μm. [file 12974_2020_1913_MOESM3_ESM.tif]

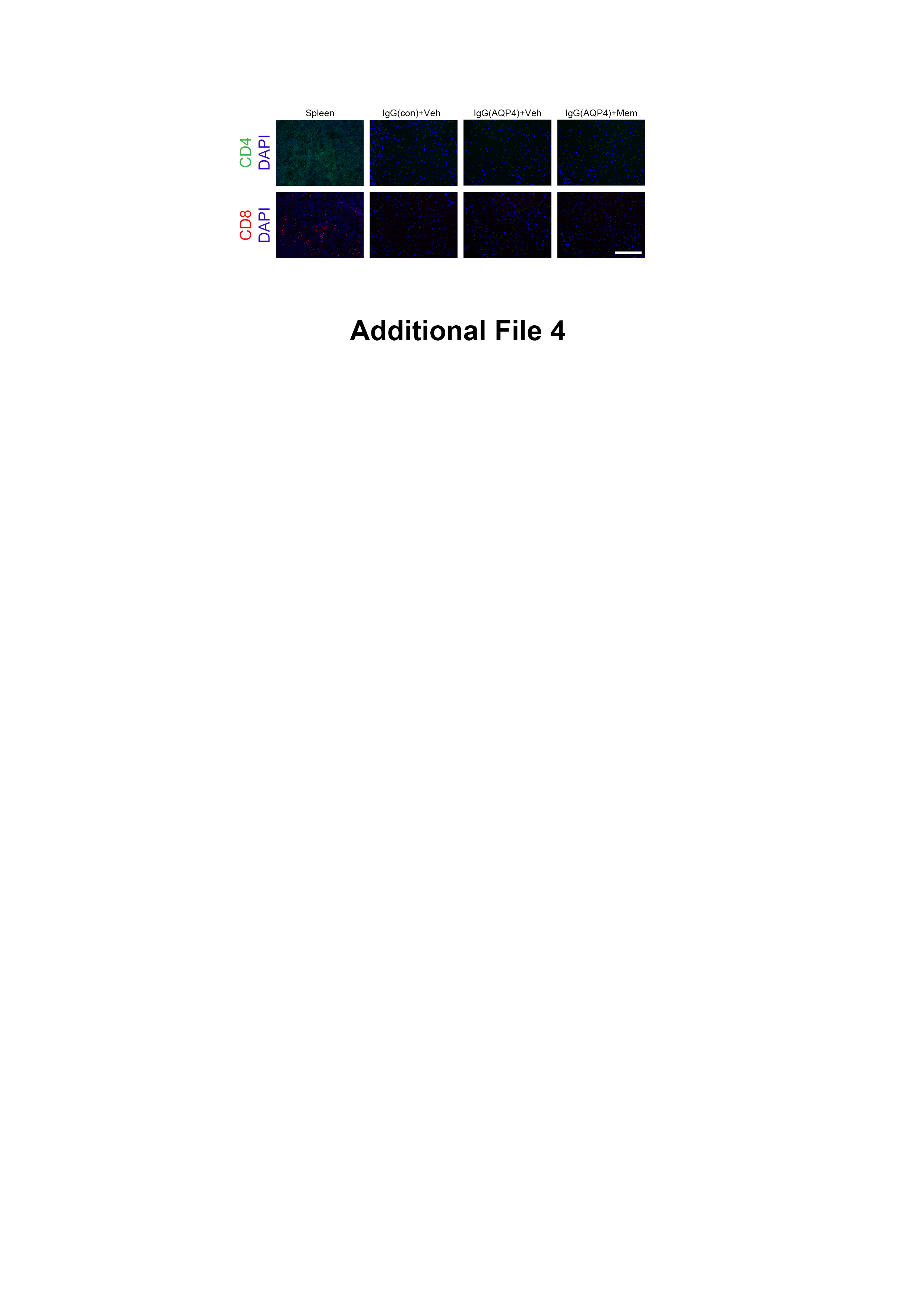

Supplement: Supplementary file 12 — Additional file 4. No T cells infiltration to the spinal cord. a Immunofluorescence staining of CD4 (T helper cell marker) and CD8 (T cytotoxic cell marker) in the spinal cords of IgG(AQP4+) mice treated with therapeutic memantine or vehicle at 8 dpi. IgG(con) mice treated with vehicle were used as a sham control. Spleens were used as a positive staining control. Sections were counterstained with DAPI. Photomicrographs are representatives of 5 animals from each group. Scale bar = 100 μm. [file 12974_2020_1913_MOESM4_ESM.tif]

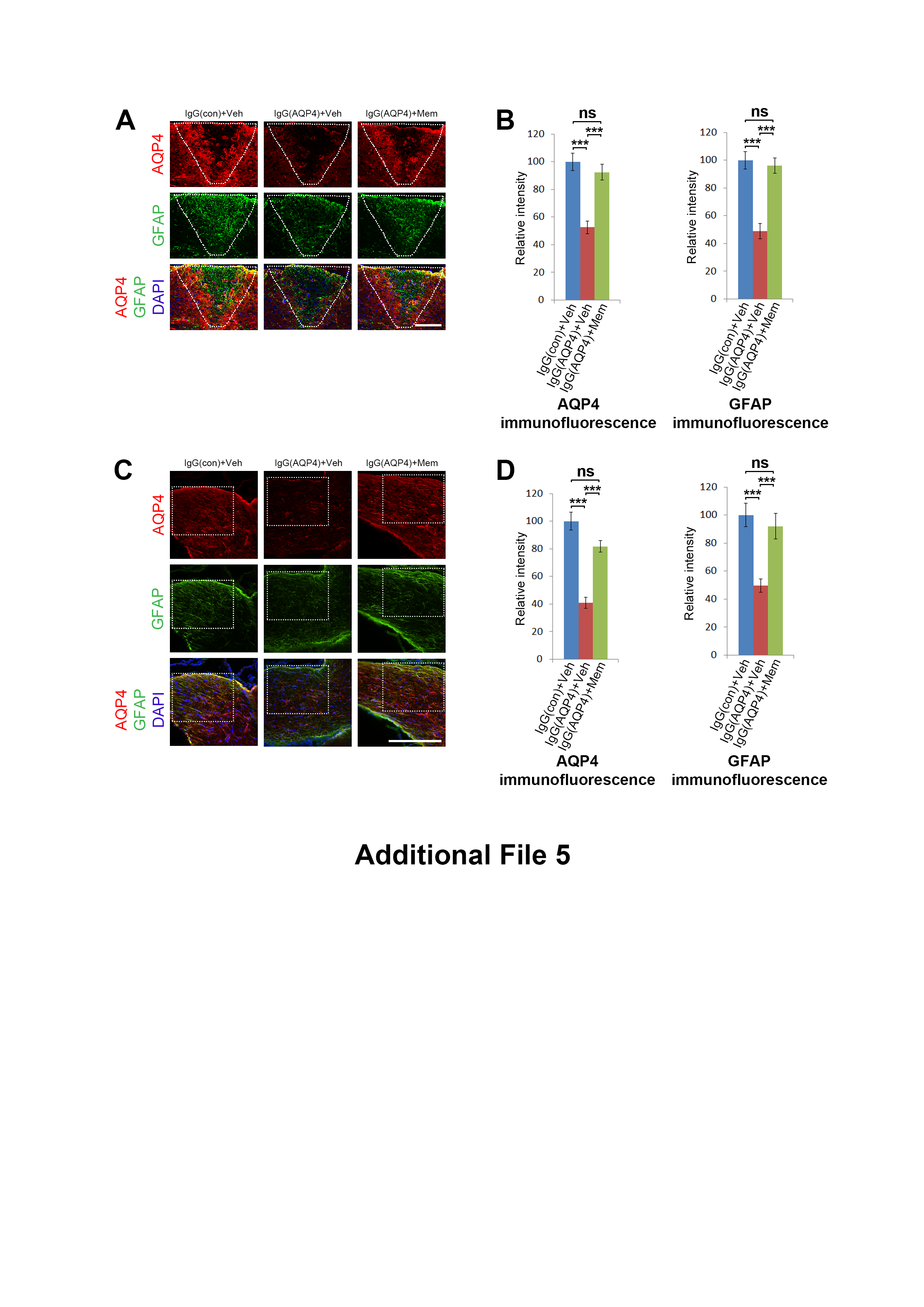

Supplement: Supplementary file 13 — Additional file 5. Memantine decreases AQP4 and GFAP loss in the area postrema and optic nerves of IgG(AQP4+) mice. a Representative photomicrographs of brain stem cross sections showing the area postrema. Double immunofluorescence staining of AQP4 and GFAP in IgG(AQP4+) mice treated with therapeutic memantine or vehicle at 8 dpi. IgG(con) mice treated with vehicle were used as a sham control. b Relative intensity of AQP4 and GFAP immunofluorescence in the area postrema. c Representative photomicrographs of longitudinal sections of the optic nerve region proximal to the optic chiasm. Double immunofluorescence staining of AQP4 and GFAP in IgG(AQP4+) mice treated with therapeutic memantine or vehicle at 8 dpi. IgG(con) mice treated with vehicle were used as a sham control. d Relative intensity of AQP4 and GFAP immunofluorescence in the optic nerve. Dotted lines demarcate the areas where quantifications of fluorescence intensities were performed. n = 3 per group. Data are mean ± SEM. One-way ANOVA with Tukey-Kramer post hoc test. ns, not significant; ***p < 0.001. Scale bar = 100 μm. [file 12974_2020_1913_MOESM5_ESM.tif]

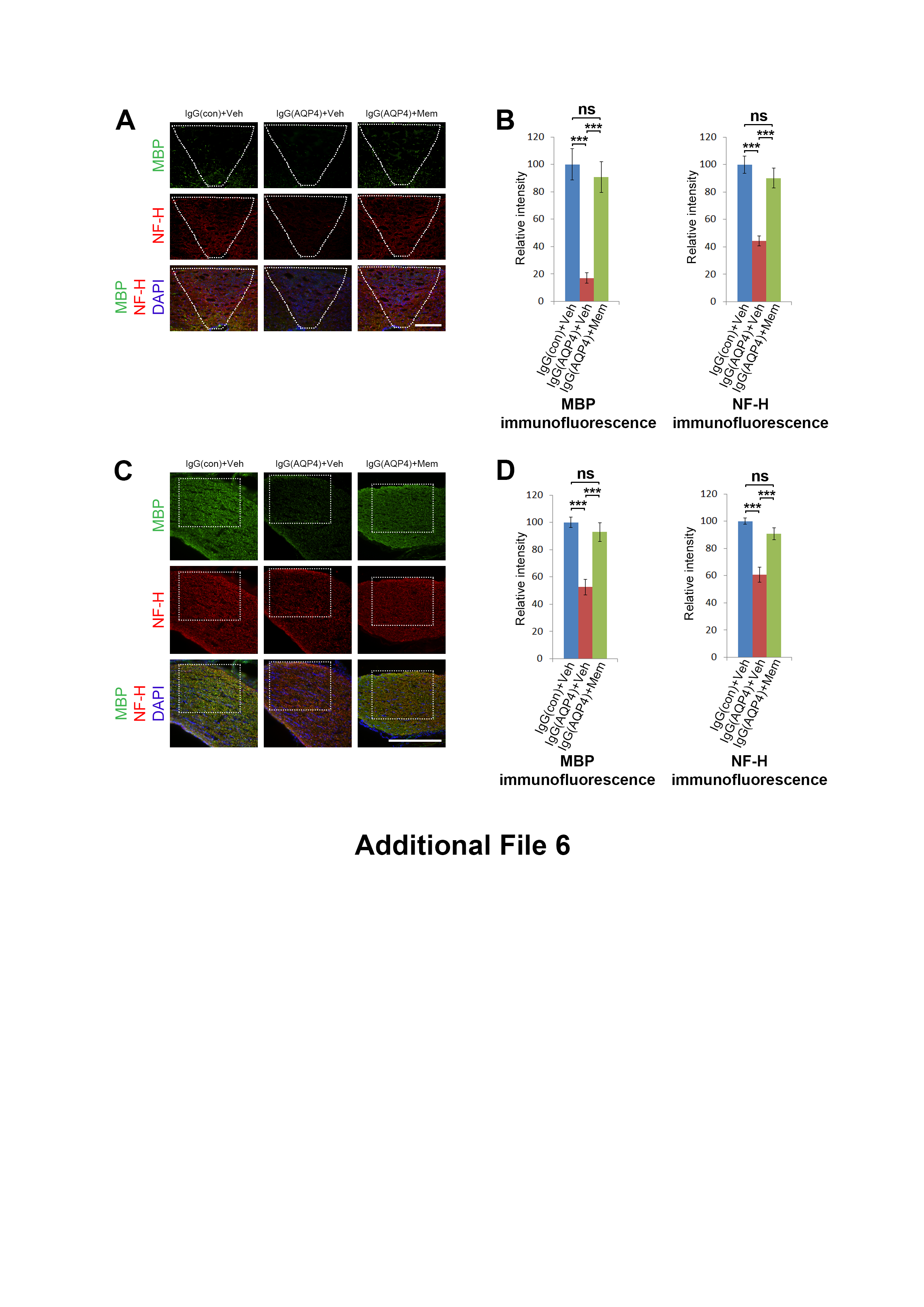

Supplement: Supplementary file 14 — Additional file 6. Memantine reduces demyelination and axonal loss in the area postrema and optic nerves of IgG(AQP4+) mice. a Representative photomicrographs of brain stem cross sections showing the area postrema. Double immunofluorescence staining of MBP and NF-H in IgG(AQP4+) mice treated with therapeutic memantine or vehicle at 8 dpi. IgG(con) mice treated with vehicle were used as a sham control. b Relative intensity of MBP and NF-H immunofluorescence in the area postrema. c Representative photomicrographs of longitudinal sections of the optic nerve region proximal to the optic chiasm. Double immunofluorescence staining of MBP and NF-H in IgG(AQP4+) mice treated with therapeutic memantine or vehicle at 8 dpi. IgG(con) mice treated with vehicle were used as a sham control. d Relative intensity of MBP and NF-H immunofluorescence in the optic nerve. n = 3 per group. Dotted lines demarcate the areas where quantifications of fluorescence intensities were performed. Data are mean ± SEM. One-way ANOVA with Tukey-Kramer post hoc test. ns, not significant; ***p < 0.001. Scale bar = 100 μm. [file 12974_2020_1913_MOESM6_ESM.tif]
